# An Activity of Thioacyl Derivatives of 4-Aminoquinolinium Salts towards Biofilm Producing and Planktonic Forms of Coagulase-Negative Staphylococci

**DOI:** 10.1155/2015/725939

**Published:** 2015-05-03

**Authors:** Robert D. Wojtyczka, Andrzej Zięba, Arkadiusz Dziedzic, Małgorzata Kępa, Danuta Idzik

**Affiliations:** ^1^Department and Institute of Microbiology and Virology, School of Pharmacy and Division of Laboratory Medicine in Sosnowiec, Medical University of Silesia, Jagiellońska 4, Sosnowiec, 41-200 Katowice, Poland; ^2^Department of Organic Chemistry, School of Pharmacy and Division of Laboratory Medicine in Sosnowiec, Medical University of Silesia, Jagiellońska 4, Sosnowiec, 41-200 Katowice, Poland; ^3^Department of Conservative Dentistry with Endodontics, Medical University of Silesia, Plac Akademicki 17, Bytom, 41-902 Katowice, Poland

## Abstract

Microorganisms present in different environments have developed specific mechanisms of settling on various abiotic and biotic surfaces by forming a biofilm. It seems to be well justified to search for new compounds enabling biofilm reduction, which is highly resistant to antibiotics. This study was thus an initial assessment of the antibacterial activity of two new quinoline derivatives of a structure of 3-thioacyl 1-methyl 4-arylaminoquinolinium salts against coagulase-negative staphylococci (CoNS) isolated from a hospital environment, in a form of both biofilms and in planktonic form. Thirty-three stains of CoNS isolated from the hospital environment (air, surfaces) and seven reference strains from the ATCC collection were selected for the study. The mean MIC value for 1-methyl-3-benzoylthio-4-(4-chlorophenylamino)quinolinum chloride (4-chlorophenylamino derivative) was 42.60 ± 19.91 *μ*g/mL, and in the case of strains subjected to 1-methyl-3-benzoylthio-4-(4-fluorophenylamino)quinolinum chloride (4-fluorophenylamino derivative) activity, the mean MIC value was 43.20 ± 14.30 *μ*g/mL. The mean concentration of 4-chlorophenylamino derivative that inhibited biofilm formation was 86.18 ± 30.64 *μ*g/mL. The mean concentration of 4-fluorophenylamino derivatives that inhibited biofilm formation was higher and amounted to 237.09 ± 160.57 *μ*g/mL. Based on the results, both derivatives of the examined compounds exhibit high antimicrobial activity towards strains growing both in planktonic and biofilm form.

## 1. Introduction

Coagulase-negative staphylococci (CoNS) constitute a considerable part of the commensal flora of human skin and mucous membranes. These microorganisms are often isolated from nosocomial infections of the bloodstream, cardiovascular system, as well as infections of the eye, ear, nose, and throat [1]. Over the recent decades, the main representative of CoNS,* Staphylococcus epidermidis*, has become a significant opportunistic pathogen due to an increasing number of specialist medical procedures, including implanted objects.* Staphylococcus epidermidis* is believed to be an important microorganism responsible for infections following surgical vascular grafts, infections after implantation of central venous catheters, heart valves, ventricular assist devices, coronary stents, neurological ventricular shunts, surgical wounds, arthroprotheses, or equipment used for fracture stabilization [2–6].

Microorganisms present in various environments have developed mechanisms of settling on various abiotic and biotic surfaces. The adhesive properties of bacteria may influence the degree of their invasiveness and the likelihood of infection. Bacteria connecting to the surface form microcolonies anchored in the extracellular matrix [7]. Bacteria present in biofilms have developed a strategy of long-lasting microecosystem, representing a nonaggressive virulence. Therefore, biofilm-related infections may last for many months, extending even for the whole life; however, they rarely directly cause death [8].

A biofilm is a cyclically maturing, three-dimensional structure composed of about 85% of extracellular matrix and of about 15% of microorganism cell aggregates. The matrix is composed of polysaccharides, proteins, enzymes, DNA, bacterial glycolipids, water, and other environmental elements [9]. Because biofilm provides a reservoir for microbial cells, its dispersion enhances the risk of chronic and persistent infections. Likewise the matrix confers a protection against drugs and has environmental promoters that induce biofilm formation and contributes to drug resistance development [10, 11]. Biofilm structure promotes the antibiotic resistance through facilitated horizontal gene transfer due to the high microbial population density. Several mechanisms have been reported to contribute an increased antimicrobial resistance in biofilm structures [11, 12], including low diffusion, transmembrane passage of antibiotics across the polysaccharide matrix, physiological changes of bacteria due to slow growth rate and starvation responses (oxygen, nutrient deprivation, or environmental stress), phenotypic change of the cells forming the biofilm, the expression of efflux pumps that decrease intracellular antimicrobial concentration, and the emergence of persister cells which are multidrug-tolerant cells that have not acquired genetic resistance [13]. These factors contribute to biofilm cells being 1000-fold more resistant to antimicrobial agents than planktonic cells [11, 14, 15]. A well-known genetic element among the staphylococci with regard to biofilm formation is the* ica* operon, which encodes a polysaccharide intercellular adhesin (PIA), which is also often called poly-N-acetyl glucosamine (PNAG) according to its chemical structure [16–18]. The operon contains the* icaADBC* genes, in addition to the* icaR* gene, which exerts a regulatory function and is transcribed in the opposite direction. Earlier studies suggested a direct link between the presence of* ica* and biofilm formation. Once this operon is activated, four proteins are transcribed, IcaA, IcaD, IcaB, and IcaC, which are necessary for the biosynthesis of PIA [17, 19, 20].* IcaA* encodes N-acetylglucosaminyltransferase, which synthesises the PIA polymer. Sole expression of* icaA* induces only low enzymatic activity, but coexpression with* icaD* significantly increases the activity due to the phenotypic expression of the capsular polysaccharide.* IcaC* is responsible for formation of long chains, and* icaB* deacetylates the poly-N-acetylglucosamine molecule [17, 20]. Expression of locus* ica* genes is regulated by various environmental factors and regulatory proteins. PIA production and its deacetylation have been considered as key factors of virulence in* S. epidermidis* [20–22].

Mature biofilms have a specific three-dimensional structure, which is described as “towers” or “mushrooms” [23]. There are canals filled with liquid between the towers, and it is believed they play a significant function in nutrient supply to the cells in the deeper biofilm layers. Mechanisms leading to canal formation and biofilm organization are not as well understood as those regulating intercellular adhesion. The results obtained from studies of* Pseudomonas aeruginosa* suggest a role in cell-cell signaling as part of a quorum-sensing system [24]. In staphylococci, expression of PIA exopolysaccharide in biofilms may to some degree contribute to biofilm formation. Various mechanisms of resistance to antibiotics are well known, such as antibiotic removal by antibiotic pumps, modification of their structure by enzymes or mutations of target sites [25, 26].

Various mechanisms of resistance of biofilm-forming strains to antibiotics are suggested. The first hypothesis is the slow or incomplete antibiotic penetration inside the biofilm structure. The second hypothesis conditions antibiotic activity on the chemical differentiation of the biofilm microenvironment. According to the third hypothesis, a subpopulation of highly protected microorganisms similar to spores is formed in biofilm structures. This hypothesis is confirmed in a study on newly formed biofilm structures, which are still too thin to present a mechanical barrier against antibiotic penetration [27, 28]. This hypothesis of spore formation by some cells living in a form of biofilm may be an explanation of lowered susceptibility of biofilm bacteria to various antibiotics, disinfection means, or a wide range of different chemical compounds [8, 25].

Modern chemotherapeutic compounds such as quinoline derivatives exhibit a wide antibacterial activity towards gram-positive bacteria such as* Staphylococcus aureus*, as well as towards gram-negative bacteria [29, 30] and also antifungal and antimalarial activity [29, 31]. Modification of the main structural fragment of a drug may lead to an improvement in its antimicrobial efficiency and strength as well as mode and direction of interaction.

The current treatment and control of biofilm is complicated, because antimicrobials have been developed against planktonically grown bacteria and microorganisms in metabolically active stage. Therefore, we have made an attempt to assess the activity of newly synthetized antibacterial agents 1-methyl-3-benzoylthio-4-(4-chlorophenylamino)quinolinum chloride (4-chlorophenylamino derivative) and 1-methyl-3-benzoylthio-4-(4-fluorophenylamino) quinolinum chloride (4-fluorophenylamino derivative) towards the strains of coagulase-negative staphylococci (CoNS), growing in a form of biofilm (BF) and in planktonic form (PF), isolated from a hospital environment. The investigated compounds were obtained as a result of an acylation of suitable derivatives of 1-methyl-4-aminoquinolinium-3-thiolates using benzoyl chloride [29, 30]. These compounds exhibit strong nucleophilic properties and easily undergo alkylation and acylation reactions on thiolate sulfur atom. The new compounds described, 4-chlorophenylamino derivative and 4-fluorophenylamino derivative, similar to 4-quinolones, are the quinoline derivatives; however their structure, compared to chinolones, is significantly different. 4-Aminoquinoline derivatives have been used as antimalarial drugs, while fluorine derivatives of 4-quinolones are an important class of antibiotics. The evaluated quinoline compounds contain the chlorine or fluoride in phenylamine substituent and not in quinoline ring as it is in case of quinolones. The examined salts are the sulfate derivatives of 4-aminoquinoline, and considering their structure, they seem to be more similar to chloroquine (used for preventing malaria from* Plasmodium vivax, P. ovale, and P. malariae*) or ammonium salt.

**Figure 1:**
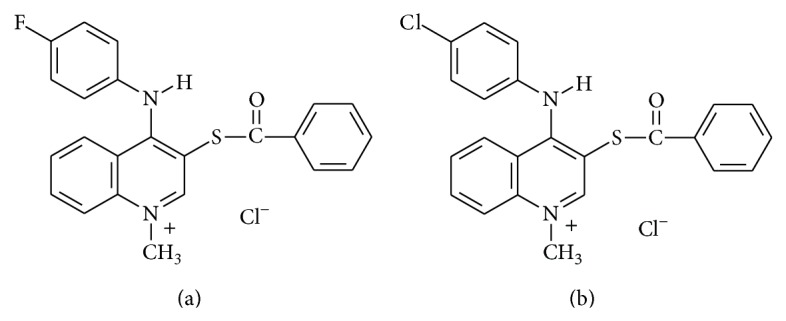
Structure of quinoline derivatives: (a) 1-methyl-3-benzoylthio-4-(4-fluorophenylamino) quinolinum chloride (4-fluorophenylamino derivative), (b) 1-methyl-3-benzoylthio-4-(4-chlorophenylamino)quinolinum chloride (4-chlorophenylamino derivative).

Quinoline is a heterocyclic aromatic organic compound with the chemical formula C_9_H_7_N. This compound forms part of the structure of quinine, the malaria remedy found in cinchona bark and known since the time of the Incas. Quinolines and their derivatives occur in numerous natural products, many of which possess interesting physiological and biological properties. Design and preparation of potential quinoline-based antibacterial agents consist partially in a molecular hybridization approach that involves the coupling of two or more groups with relevant biological properties [32].  Figure 1 presents the structure of two examined compounds.

The search for new antibacterial compounds continues and other quinolines with similar structures may become available as efficient antibiotics against resistant microorganisms. The key reason of all these structural changes of quinoline derivatives was to improve physicochemical parameters of quinoline molecules, which may lead to an enhanced fit into the binding site. As the hospital strains become increasingly resistant to the standard antibiotics, there is an urgent need to understand the molecular mechanisms for new drug action and resistance so that novel antibacterial drugs can be designed. A number of modified quinolines compounds show some promise in this regard.

## 2. Material and Methods

### 2.1. Bacterial Strain

Thirty-three isolated of coagulase-negative staphylococci obtained from a hospital environment (surfaces, air) and eight reference strains from the ATCC collection (*S. epidermidis* ATCC 12228,* S. epidermidis* ATCC 35984,* S. saprophyticus* ATCC 15305,* S. hominis* ATCC 27844,* S. haemolyticus* ATCC 29970,* S. capitis* subsp.* capitis* ATCC 35661,* S. warneri* ATCC 49454, and* S. lugdunensis* ATCC 49567) were selected for the study. The species of the strains were identified according to standard methodology with the use of biochemical APIStaph tests (bioMerieux, France) and stored for further analysis on TBE medium with the addition of 20% glycerol (Sigma) at −20°C reference strains from the ATCC collection were obtained from LCG Standards (Lomianki, Poland).

### 2.2. Investigation of Biofilm Production by the Congo Red Agar (CRA) Method

Phenotypic characterization of biofilm production was performed by culturing the CoNS isolates on CRA plates as described by Freeman et al. [33]. A special medium was prepared of brain-heart infusion broth (BHI-BTL Poland) supplemented with 5% sucrose and Congo red. Plates were inoculated and incubated aerobically for 24 to 48 hours at 37°C. According to the authors, biofilm producers form black, crusty colonies on CRA, whereas nonproducers form red colonies. A darkening of the colonies with the absence of a dry crystalline colonial morphology indicated an intermediate result.

### 2.3. Biofilm Formation Studied by Microtiter Plate Assay (MP)

We performed the microtiter plate assay described by Christensen et al. [34] with modifications. A suspension equivalent to McFarland 0.5 turbidity standard was prepared in Muller-Hinton broth (MHB-BTL, Poland) for each strain. The accuracy of bacterial counts in the suspension was confirmed by serial dilution in log steps. Aliquots of 100 *μ*L from each bacterial suspension were inoculated onto 96-well tissue microtiter plates. These were incubated at 37°C for 24 h in a normal atmosphere. The medium was then removed, and the wells were washed three times with phosphate-buffered saline (PBS, pH 7.2) to remove free-floating “planktonic” bacteria. An amount of 150 *μ*L of 0.1% crystal violet (Sigma, USA) was added to each well and left for 30 min at room temperature. The dye was removed, and this was followed by five washes with sterile deionized water. The preparations were destained with 200 *μ*L of 95% isopropanol in 1 M HCl for 5 min. Finally, 100 *μ*L of coloured isopropanol from each sample was transferred to another microtiter plate. The optical density (OD) of the suspension was measured at a wavelength of 490 nm with a Multitec SX microplate reader.

The ODs obtained were compared with those of the negative control (well without bacterial inoculum). We considered the OD > 0.17 to be positive. All the strains were tested four times, and the average value for each sample was calculated. The mean* A*_490_  ± SD values are presented.

The isolates were classified into two categories: nonadherent, optical density to or lower than 0.17 and adherent, optical density higher than 0.17. When the cut-off corresponded to nonadherent, the isolates were classified as negative and as positive when the cut-off corresponded to adherent.

### 2.4. Detection of* icaA* and* icaD* Genes Specific for Biofilm Production in* S. epidermidis* Strains

All isolates were stored at −80°C pending analysis and were subcultured on blood agar plates and checked for purity prior to DNA preparation. An Isolate Genomic DNA Mini Kit (BLIRT S.A., Poland) was used to isolate DNA from strains with the following modifications. Pure DNA was stored at −20°C.

PCR was used to detect the presence of* icaA* and* icaD*. The primer sequences and predicted product lengths for* icaA* and* icaD* were described by Ziebuhr et al. [35] and de Silva et al. [36]. The PCR reactions were performed in separate reactions using a 10x PCR RED master mix kit. The PCR cycling conditions used were 30 cycles of 30 s of denaturation at 95°C and 3 min of elongation at 72°C for all reactions, with an annealing for 1 min at 60°C (*icaA*) or 59°C (*icaD*). PCR was performed by using an MJ Mini Personal thermal cycler (BIORAD, Germany).

The PCR products were electrophoresed through 1.5% agarose gels and checked for size against molecular weight markers using 1 Kb HypeLadderIV (BLIRT S.A., Poland).

### 2.5. Examination of Antistaphylococcal Activity of 1-Methyl-4-aminoquinolinium-3-thiolates

Determination of minimum inhibitory concentrations (MIC) of microorganisms was performed using the method of microdilutions on 96-well plates according to obligatory methodology [37].

The MIC value was determined by an incubation of the examined strains in 96-well plates for 20 hours at 37°C. Bacterial inocula were prepared in 0.9% sodium chloride from fresh cultures up to the moment of 0.5 McFarland's turbidity. Next, the inocula were diluted (1 : 100) in sterile Mueller-Hinton broth medium just before addition to the plates. The microorganisms were exposed to a serial dilution of 4-chlorophenylamino derivative and 4-fluorophenylamino derivative in the range from 1000 to 1 *μ*g/mL. In addition, the values of minimum and maximum MIC as well as MIC_50_ and MIC_90_ were determined. The examinations were conducted both for the strains forming biofilm and those not forming it.

### 2.6. Inhibition of Staphylococcal Biofilm Formation by 1-Methyl-4-aminoquinolinium-3-thiolates

The 1-methyl-4-aminoquinolinium-3-thiolates were tested for their potential to prevent biofilm formation of a biofilm producing* S. epidermidis* strain. The evaluation of minimum biofilm inhibiting concentrations (MBIC) was done colorimetrically from the metabolic activity of surviving CoNS strains cells treated with new quinoline compounds [38, 39]. The presence of viable cells in biofilm was tested using 3-(4,5-dimethylthiazol-2-yl)-2,5-diphenyltetrazolium bromide (MTT). This “static,” indirect viability assay measures enzymatic activity in actively respiring cells and is based on the formation of insoluble purple formazan due to the reduction of MTT by respiratory reductase of living staphylococcal cells. Therefore, it is a measure of cell viability and/or relative numbers of viable cells. The minimum biofilm inhibitory concentration (MBIC) is defined as the lowest concentration of an antimicrobial agent required to inhibit the formation of biofilms. Nonbiofilm-forming clinical isolate* S. epidermidis* was used as negative control. The bioassay was performed in triplicate.

For assessing the biofilm growth inhibition effects of the 4-chlorophenylamino derivative and 4-fluorophenylamino derivative, they were added to the growth medium at the time of in oculation and the cells were allowed to form biofilm.

An aliquot of twofold serial dilutions (200 *μ*L) was prepared in the 96-well microtiter plate containing tryptic soy broth (TSB, BTL, Łódź, Poland) supplemented with 2% D-glucose (TSBGlc), with final concentrations of 4-chlorophenylamino derivative ranging from 1000 *μ*g/mL to 1 *μ*g/mL for 4-fluorophenylamino derivative. Bacterial suspensions (100 *μ*L; 5 × 105 CFU/mL, final concentration) were then transferred into the plate. TSBGlc containing 0.2% DMSO was employed as a negative control. TSBGlc without the extract was used as the nontreated well and the medium with each concentration of the extracts was used as the blank control [40].

After incubation in 37°C for 24 hours the inhibitory effect was evaluated using MTT reduction assay. The supernatant was discarded and replaced with 200 *μ*L of PBS supplemented with 50 *μ*g MTT (Sigma-Aldrich, USA). The formazan crystals were dissolved in dimethyl sulfoxide (DMSO, Sigma-Aldrich, USA), and the absorbance was determined at 570 nm (OD = 570 nm) with microplate reader (Thermo Electron Corp., Finland).

### 2.7. Statistical Analysis

All examinations of MIC and biofilms were performed four times, and the results were expressed as mean values ± standard deviation (SD). Results from correlation assays were submitted to the *U* Mann-Whitney nonparametric comparing biofilm formation status and* ica* status, MP test, and CRA plate test. Results were considered significant when *P* < 0.05. For comparison of the MIC and MBIC results, the respective 50th percentile values (MIC_50_ and MBIC_50_) and 90th percentile values (MIC_90_ and MBIC_90_) were calculated, and the ranges were compared. Statistical analyses were performed using Statistica 10.0 PL (StatSoft).

## 3. Results

Analyses of the antimicrobial activity of 4-chlorophenylamino derivatives and 4-fluorophenylamino derivatives were conducted on 40 strains of coagulase-negative staphylococci, of which 33 strains were isolated from a hospital environment (air, surfaces) and seven reference strains were derived from the American Type Culture Collection. Among 33 of the identified CoNS environmental strains, 19 were identified as* S. epidermidis*, 5 strains as* S. haemolyticus*, 3 strains as* S. warneri*, 2 strains as* S. cohnii* subsp*. cohnii,* and 1 strain as* S. saprophyticus*,* S. kloosii*,* S. cohni* subsp*. urealyticum*, and as* S. capitis* subsp*. capitis*. Among the ATCC strains, the following were used in the study:* S. epidermidis* ATCC 12228,* S. saprophyticus* ATCC 15305,* S. hominis* subsp*. hominis* ATCC 27844,* S. haemolyticus* ATCC 29970,* S. capitis* subsp*. capitis* ATCC 35661,* S. warneri* ATCC 49454, and* S. lugdunensis* ATCC 49567.

By assessing the ability of biofilm formation using the CRA method, characteristic black crusty colonies were obtained only in the case of two (5%) strains, and intermediate colonies, grey ones in three (7.5%) cases. The examined reference strains did not exhibit an ability for biofilm formation using the CRA method (Table 1).

Among 40 strains of coagulase-negative staphylococci analyzed using the MP method, 11 (27.5%) strains were classified as forming a biofilm (*A*_490_ > 0.17). The other 22 examined strains and 7 reference strains did not form biofilms (Table 1). Among the 11 strains forming biofilms, 10 (90.9%) belonged to* S. epidermidis* species, while 1 strain (9.1%) was identified as* S. kloosii*.

After examining the presence of* icaA* and* icaD* genes in* S. epidermidis* isolated strains, the* icaAD* gene was noted in 11 strains (57.9%) (Table 1). Analyzing the results of an assessment of an ability of biofilm formation using CRA, MP methods, and determination of the presence of* icaA* and* icaD* genes, it may be concluded that 9 (90%) out of 10* S. epidermidis* strains exhibiting phenotypical ability of biofilm formation using the MP method demonstrated the presence of at least one of these genes (*icaA*). In this one analyzed case, this may suggest the possibility of biofilm formation independent of the* icaADBC* operon. In one case, the presence of the* icaA* gene did not induce the phenotypical presence of biofilm in any of the methods used. Statistical analysis by the Kruskal-Wallis test showed a correlation among the phenotypical presence of biofilm and* ica* status (*P* = 0.0017).

Analyses of the MIC values for 40 strains of coagulase-negative staphylococci (Tables 2 and 3) showed a mean MIC value for 4-chlorophenylamino derivative of 42.60 ± 19.91 *μ*g/mL, with a minimum value of 16 *μ*g/mL and maximum of 64 *μ*g/mL. In the case of the strains subjected to 4-fluorophenylamino derivative, the mean MIC value was 43.20 ± 14.30 g/mL, the minimum value was 16 *μ*g/mL, and the maximum was 64 *μ*g/mL. The values of MIC_50_ and MIC_90_ for all examined strains were 46 *μ*g/mL and 64 *μ*g/mL, respectively, and were the same for 4-chlorophenylamino derivative and 4-fluorophenylamino derivatives.

Assessing the ability of biofilm inhibition by 4-chlorophenylamino derivative and 4-fluorophenylamino derivative, 11 strains exhibiting such ability phenotypically were selected for analysis using the MP method (Table 4). The analysis showed that biofilms grown in the presence of compounds had significantly lower activity as compared to an inhibitor control.

The mean concentration of 4-chlorophenylamino derivative inhibiting biofilm formation for the examined strains of coagulase-negative staphylococci was 86.18 ± 30.64 *μ*g/mL, with a minimum of 40 *μ*g/mL and maximum of 128 *μ*g/mL. In the case of 4-fluorophenylamino derivatives the mean concentration inhibiting biofilm formation was higher and amounted to 237.09 ± 160.57 *μ*g/mL, while the minimum and maximum values were 56 *μ*g/mL and 448 *μ*g/mL, respectively (*P* < 0.001). Values of MBIC_50_ and MBIC_90_ for 4-chlorophenylamino derivative were 96 *μ*g/mL and 128 *μ*g/mL. In the case of 4-fluorophenylamino derivatives the MBIC_50_ reached a value of 192 *μ*g/mL, and the MBIC_90_ value was 448 *μ*g/mL.

## 4. Discussion

The results of our study demonstrated the presence of the* icaA* and/or* icaD* genes and subsequent biofilm production in most* S. epidermidis* isolates (sensitivity 0.909; specificity 0.724). Taking into account the fact that biofilm structures are the main factor of virulence involved in infections with coagulase-negative staphylococci [1], it seems to be essential to determine their antibiotic activity considering infections caused by these microorganisms occur both in planktonic forms and those forming a biofilm. Scanning electron microscopy demonstrated that biofilm is visible as soon as after 24 hours of bacterial adhesion to the surface [41]. According to some observations, 99% of bacteria live in a form of bacterial biofilm, and only 1% in a free form not attached to the surface, for example, in body liquids [7].

Numerous studies were carried out with the attempt to compare the susceptibility of bacteria growing in a form of biofilm on selected antibiotics, as well as their equivalents in planktonic form; however the results were not validated and/or were unequivocal [42, 43]. Bacteria growing in a form of biofilm are from 100- up to 1000-fold more resistant than their planktonic forms [42–44].

An assessment of the bactericidal activity of six selected antibiotics on the planktonic and biofilm forms of three* S. epidermidis* strains and three* S. aureus* strains was conducted by Nishimura et al. [41]. In the case of clarithromycin, cefotaxime, erythromycin, and benzylpenicillin, the planktonic forms of the* S. epidermidis* strains demonstrated susceptibility to antibiotic concentrations below 0.5 *μ*g/mL, while in the case of vancomycin and cefmetazole this value was estimated at 1 *μ*g/mL. Conducting a similar analysis of biofilm-forming bacteria, Nishimura et al. demonstrated their susceptibility in concentrations not lower than 512 *μ*g/mL to 1024 *μ*g/mL [41].

The activity of various antibiotics including daptomycin, vancomycin, gentamicin, and rifampicin applied as a monotherapy and combined polytherapy against two clinical MRSA strains forming biofilm l was investigated by LaPlante and Woodmansee [45]. They noted that daptomycin and rifampicin had only slight differences in the ratio between MIC and MBEC. In the case of vancomycin, the MIC values for two examined strains forming biofilms were 2 *μ*g/mL and 4 *μ*g/mL, respectively, while the MBEC values for the same strains were considerably higher and amounted to 128 mg/L and 64 mg/L, respectively. These strains were more susceptible to gentamicin with even higher differences between the MIC and MBEC values, and they were estimated as 0.5 *μ*g/mL and 256 mg/L, respectively, for both strains.

Analysis of the susceptibility of eight* S. aureus* and CoNS strains forming biofilms with respect to azithromycin in a form of eye solution was conducted by Wu et al. [46]. The MIC values for eight examined strains ranged from 0.75 *μ*g/mL to >256 *μ*g/mL, and both strains were susceptible and resistant to azithromycin. Analyses of the susceptibility of biofilm forms of these strains to azithromycin in a form of eye solution (concentration of 0.25–1.0%) showed an inhibition of biofilm formation irrespective of the strains' susceptibility or resistance to azithromycin.

An analysis of various antibiotic (cefazolin, vancomycin, dicloxacillin, tetracycline, and rifampicin) activity mechanisms towards six strains of coagulase-negative staphylococci was conducted in turn by Cerca et al. [44]. When the target site of the antibiotic activity was the cell membrane, a reduction in biofilm formation was observed irrespective of the size of the antibiotic particle. Moreover, when the antibiotic target site was RNA or protein synthesis, a similar activity was noted for planktonic and biofilm forms. They concluded that phenotypical antibiotic resistance of bacterial cells in a biofilm is predominantly related to the mechanism of antibiotic activity.

A significant variation in bactericidal activity between planktonic and biofilm forms of bacteria was also reported by El-Banna et al. [47]. Analyzing eight strains of staphylococci (three strains of* S. aureus* and five CoNS strains), they showed that, considering ciprofloxacin, the bactericidal value represented by the MIC was significantly different. For biofilm-forming strains, the MIC values were from 2- to 512-fold higher than those obtained for planktonic forms. These authors also obtained similar results by analyzing the activity of gentamicin towards the same bacterial strains. In the case of amoxicillin with clavulanic acid, the value of MBC for biofilm forms was 4 to 64 times higher than the MBC results obtained for bacteria growing in planktonic form.

Comparing the minimum concentration inhibiting microorganism growth and the minimum concentration inhibiting biofilm formation for the examined CoNS strains, we demonstrated that in the case of 4-chlorophenylamino derivative the MIC values are only about 2-fold higher, and for 4-fluorophenylamino derivative they were approximately 6- to 10-fold higher (*P* = 0.009). Therefore, both derivatives of the examined compounds demonstrate high activity with respect to strains growing in a biofilm, and a clear predominance is demonstrated for 4-chlorophenylamino derivative.

The molecular basis of the action of these new compounds is not completely understood, but they are thought to interfere with bacterial DNA synthesis. Structure-activity relationship studies revealed that the antimicrobial activity in this heterocyclic class of quinoline molecules depends on the nature of the peripheral substituents and their spatial relationship within the quinoline skeleton. This type of quinolines may inhibit DNA synthesis by promoting cleavage of bacterial DNA in the DNA-enzyme complexes of type II topoisomerase DNA gyrase and the topoisomerase IV, resulting in rapid bacterial death [32].

The examined quinoline derivatives were able to inhibit biofilm formation, detach existing biofilms, and kill bacteria in biofilms of selected CoNS strains. Importantly, biofilms were similarly as sensitive as their planktonic counterparts, probably due to the dual activity of tested compounds on existing biofilms. MBIC values of thioacyl derivatives of 4aminoquinolinium salts against hospital isolates are being documented for the first time in this study which outlines a very sturdy basis for future investigations in pursuit to discover new anti-infectious agents. Further study is advised to elucidate the complex mode of action of newly synthetized compounds against biofilms of CoNS, particularly* S*.* epidermidis* and other clinically relevant microbes.

## 5. Conclusions

The administration of suboptimal chemotherapeutic concentrations, active only for planktonic forms, not the main infection factor, may be inefficient for pharmacological therapy. Therefore, it seems to be viable and purposeful to search for such antibacterial agents with similar activities towards planktonic and biofilm forms of bacteria. Considering the examined compounds, we conclude that only 4-chlorophenylamino derivative demonstrates such abilities. However, further analysis not only of antistaphylococcal activities and pharmacokinetics but also of drug interactions and chronic cumulative toxicities due to long-term administration is needed prior to clinical application of these drugs.

## Figures and Tables

**Table 1:** Phenotypic and genotypic characterization data from 41 CoNS strains.

Strain number	Bacterial species	Biofilm status	MP test^∗1^ (*A*_490_ ± SD)	CRA plate test^∗∗2^	Presence of *icaA/icaD *gene^∗∗∗2^
1	*S. saprophyticus *	Biofilm-negative	0.07 ± 0.01	r	ND
2	*S. klosii *	Biofilm-positive	0.44 ± 0.08	b	ND
4	*S. cohnii *subsp*. urealyticum *	Biofilm-negative	0.11 ± 0.01	r	ND
5	*S. haemolyticus *	Biofilm-negative	0.08 ± 0.02	r	ND
14	*S. cohnii *subsp*. cohnii *	Biofilm-negative	0.10 ± 0.04	g	ND
24	*S. epidermidis *	Biofilm-positive	0.48 ± 0.06	r	+/−
25	*S. epidermidis *	Biofilm-positive	0.44 ± 0.10	r	+/−
26	*S. epidermidis *	Biofilm-positive	0.61 ± 0.03	r	+/+
27	*S. epidermidis *	Biofilm-negative	0.07 ± 0.02	r	−/+
28	*S. epidermidis *	Biofilm-negative	0.08 ± 0.01	r	−/−
32	*S. warneri *	Biofilm-negative	0.06 ± 0.01	r	ND
33	*S. epidermidis *	Biofilm-positive	0.19 ± 0.01	r	+/−
53	*S. epidermidis *	Biofilm-negative	0.13 ± 0.01	r	+/+
62	*S. cohnii *subsp*. cohnii *	Biofilm-negative	0.07 ± 0.02	g	ND
64	*S. epidermidis *	Biofilm-negative	0.08 ± 0.02	r	−/−
65	*S. epidermidis *	Biofilm-positive	0.27 ± 0.08	r	−/−
76	*S. epidermidis *	Biofilm-positive	0.47 ± 0.05	r	+/−
78	*S. epidermidis *	Biofilm-positive	0.53 ± 0.10	r	+/−
84	*S. epidermidis *	Biofilm-negative	0.09 ± 0.02	r	−/−
90	*S. epidermidis *	Biofilm-negative	0.07 ± 0.01	g	−/−
91	*S. epidermidis *	Biofilm-positive	0.45 ± 0.04	b	+/+
93	*S. haemolyticus *	Biofilm-negative	0.05 ± 0.00	r	ND
95	*S. epidermidis *	Biofilm-negative	0.10 ± 0.05	r	−/−
96	*S. haemolyticus *	Biofilm-negative	0.11 ± 0.02	r	ND
97	*S. epidermidis *	Biofilm-positive	0.46 ± 0.01	r	+/+
99	*S. epidermidis *	Biofilm-positive	0.55 ± 0.05	r	+/+
101	*S. haemolyticus *	Biofilm-negative	0.06 ± 0.01	r	ND
102	*S. epidermidis *	Biofilm-negative	0.06 ± 0.00	r	−/+
103	*S. capitis *subsp*. capitis *	Biofilm-negative	0.14 ± 0.03	r	ND
105	*S. haemolyticus *	Biofilm-negative	0.10 ± 0.03	r	ND
107	*S. epidermidis *	Biofilm-negative	0.07 ± 0.02	r	−/−
125	*S. warneri *	Biofilm-negative	0.08 ± 0.03	r	ND
139	*S. warneri *	Biofilm-negative	0.10 ± 0.04	r	ND
12228	*S. epidermidis* ATCC 12228	Biofilm-negative	0.11 ± 0.02	r	−/−
35984	*S. epidermidis *ATCC 35984	Biofilm-positive	0.56 ± 0.11	b	+/+
15305	*S. saprophyticus* ATCC 15305	Biofilm-negative	0.09 ± 0.03	r	ND
27844	*S. hominis *ATCC 27844	Biofilm-negative	0.06 ± 0.01	r	ND
29970	*S. haemolyticus* ATCC 29970	Biofilm-negative	0.08 ± 0.03	r	ND
35661	*S. capitis *subsp*. capitis* ATCC 35661	Biofilm-negative	0.08 ± 0.01	r	ND
49454	*S. warneri* ATCC 49454	Biofilm-negative	0.08 ± 0.02	r	ND
49576	*S. lugdunensis* ATCC 49576	Biofilm-negative	0.06 ± 0.01	r	ND

CRA: type of growth on Congo Red Agar medium, r: red colonies, b: black colonies, and g: grey colonies;

*icaA/D*: (+) presence of the gene, (−): absence of the gene, and ND: not detection.

Correlation between biofilm status and ^∗1^MP test *r* = *P* < 0.001; ^∗∗2^CRA plate test *r* = 0.13 (*P* = 0.785); ^∗∗∗2^ *ica* status *r* = 0.43 (*P* = 0.015). Results were considered significant when *P* < 0.05; ^1^ *U* Mann-Whitney nonparametric test; ^2^Rang Spearman test.

**Table 2:** Mean MIC values for CoNS strains subjected to an activity of 4-chlorophenylamino derivative and 4-fluorophenylamino derivative (*μ*g/mL).

Bacterial species (number of strains)	4-Chlorophenylamino derivative MIC ± SD	4-Fluorophenylamino derivative MIC ± SD
*S. epidermidis* (19)	43 ± 7.13	41 ± 6.41
*S. haemolyticus* (5)	42 ± 8.75	39 ± 20.65
*S. warneri* (3)	31 ± 8.83	43 ± 0.00
*S. cohnii *subsp*. cohnii* (2)	28 ± 4.62	36 ± 13.90
*S. klosii *(1)	48 ± 18.48	16 ± 0.00
*S. cohnii *subsp*. urealyticum* (1)	64 ± 0.00	56 ± 16.00
*S. saprophyticus* (1)	24 ± 9.24	32 ± 0.00
*S. capitis *subsp*. capitis* (1)	48 ± 18.48	48 ± 18.48
*S. epidermidis * ATCC 12228	28 ± 8.00	64 ± 0.00
*S. epidermidis *ATCC 35984	64 ± 0.00	48 ± 18.48
*S. saprophyticus* ATCC 15305	24 ± 9.24	56 ± 16.00
*S. hominis *ATCC 27844	64 ± 0.00	48 ± 18.48
*S. haemolyticus* ATCC 29970	64 ± 0.00	56 ± 16.00
*S. capitis *subsp*. capitis* ATCC 35661	48 ± 18.48	48 ± 18.48
*S. warneri* ATCC 49454	64 ± 0.00	48 ± 18.48
*S. lugdunensis* ATCC 49567	48 ± 18.48	64 ± 0.00

**Table 3:** Statistical analysis of obtained MIC values (*μ*g/mL) 4-chlorophenylamino derivative and 4-fluorophenylamino derivative with respect to CoNS strains depending on growth form.

	Biofilm-negative strains MIC ± SD (*µ*g/mL) *n* = 29	Biofilm-positive strains MIC ± SD (*µ*g/mL) *n* = 12	*P*
4-Chlorophenylamino derivative	40.14 ± 15.73	49.09 ± 15.19	0.016
4-Fluorophenylamino derivative	45.52 ± 13.79	37.09 ± 14.43	0.018

Results were considered significant when *P* < 0.05; *U* Mann-Whitney nonparametric test.

**Table 4:** Values of minimum biofilm inhibitory concentration (MBIC) of the examined strains.

Bacterial species (number of strains)	4-Chlorophenylamino derivative MBIC ± SD (*μ*g/mL)	4-Fluorophenylamino derivative MBIC ± SD (*μ*g/mL)
*S. klosii *(1)	44 ± 24.00	56 ± 16.00
*S. epidermidis* (9)	100 ± 27.91	283 ± 96.72
*S. epidermidis* ATCC 35984	128 ± 0.00	384 ± 147.80
